# Power-law intermittency in the gradient-induced self-propulsion of colloidal swimmers†

**DOI:** 10.1039/d4sm00603h

**Published:** 2024-06-05

**Authors:** Nick Oikonomeas-Koppasis, Stefania Ketzetzi, Daniela J. Kraft, Peter Schall

**Affiliations:** a Institute of Physics, University of Amsterdam Science Park 904 P.O. Box 94485 1090 GL Amsterdam The Netherlands n.oikonomeaskoppasis@uva.nl; b Soft Matter Physics, Huygens-Kamerlingh Onnes Laboratory, Leiden University P.O. Box 9504 2300 RA Leiden The Netherlands

## Abstract

Active colloidal microswimmers serve as archetypical active fluid systems, and as models for biological swimmers. Here, by studying in detail their velocity traces, we find robust power-law intermittency with system-dependent exponential cut off. We model the intermittent motion by an interplay of the field gradient-dependent active force, which depends on a fluid gradient and is reduced when the swimmer moves, and the locally fluctuating hydrodynamic drag, that is set by the wetting properties of the substrate. The model closely describes the velocity distributions of two disparate swimmer systems: AC field activated and catalytic swimmers. The generality is highlighted by the collapse of all data in a single master curve, suggesting the applicability to further systems, both synthetic and biological.

## Abstract 2

Active colloidal microswimmers serve as archetypical active fluid systems, and as models for biological swimmers.

## Graphical abstract

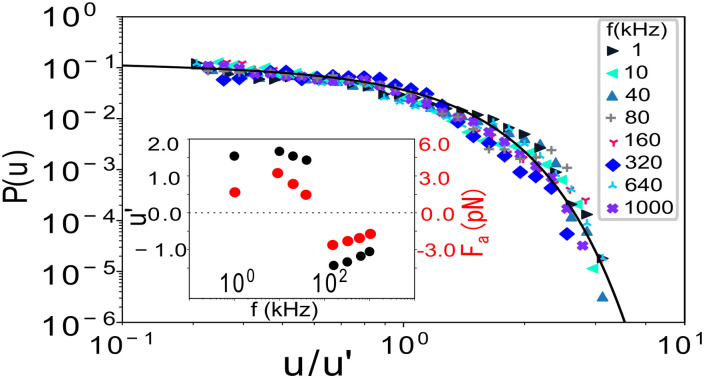

Artificial swimmers have been the focus of a wide variety of research, with increasing interest in fields such as smart drug delivery,^1^ cargo transport in micro or nano-systems,^2^ as well as the study of collective behavior in larger sytems^3^ in active matter. There are also many examples of microswimmers in nature. Spanning across five orders of magnitude in size, the dynamics of active motion is used to understand the contagion of nanometer-sized viruses,^4^ the motility patterns of micrometer-sized bacteria,^5^ and can even be extended to larger organisms such as salmon swimming against currents.^6^

The common feature of all these systems is their ability to draw energy from their environment to perform directed motion.^7,8^ A simple realization of an experimental model to study such active motion are Janus colloids, exhibiting two or more distinct surfaces, leading to a chemical gradient of solutes properties, propelling the particle. The mechanism with which the gradients are induced can vary from preferential catalytic reactions^9^ to local binary solvent demixing induced by light absorption,^10^ to AC field application.^11^

In all cases, the solute gradient leads to self propulsion of the particle, which can itself affect the solute gradient by advection.^12,13^ This influence of the particle motion on the solute gradient has been discussed in hydrodynamic frameworks;^12–15^ if the particle motion-related advection becomes of the order of the solute diffusion, the advection will strongly distort the solute gradient that would establish for the particle at rest. Furthermore, the artificial swimmers typically self-propel close to a substrate that exhibits a characteristic drag on the moving particle, and to understand the full mechanism of swimming, one has to take into account the hydrodynamic interaction of particle and substrate as well.^16,17^

Experimental insights into these mechanisms, however, are limited, despite their importance for understanding the full dynamics of propulsion. In experiments, it is standard practice to report the average swimming velocity^11,17^ or control of the swimming direction^10^ as a function of the driving force parameters such as fuel concentration or frequency of applied AC fields. Detailed studies of the propulsion dynamics are scarce. Some intricate dynamics of different systems have been reported, some system specific, and others more generalized,^16^ but despite sharing multiple common traits, different active systems are usually being discussed separately. An exception to this is the study of bacteria swimming by Lisicki *et al.*,^18^ in which the authors collected data of swimming micro-organisms and grouped them according to the swimming mechanism. However, the commonalities of many of these systems in terms of their physical hydrodynamic mechanism of active swimming have not been addressed, masking any underlying dynamics of active motion.

In this paper, we study in detail the time-dependent propagation of colloidal swimmers and elucidate underlying principles of their swimming mechanism. We investigate two disparate colloidal swimmer systems: a system activated by an AC field, and a system of catalytic swimmers, activated by the creation of a solute gradient *via* a catalytic reaction that propels the particle. We uncover surprising intermittency in the active swimmer motion, yielding velocity distributions with robust power laws in both systems with active force-dependent exponential cut-off. We show that this intermittency results from a competition between the self-propulsion, restricted by the diffusion-limited replenishment of the fuel gradient driving the motion, and the hydrodynamic drag, modulated due to wetting inhomogeneities of the nearby wall. The generality of these findings is demonstrated by collapse of all velocity distributions into a single master curve. These results, revealing an intriguing coupling mechanism behind the self-propelling motion, may apply not only to synthetic colloidal swimmers, but also more generally to biological swimmers driven by a gradient, such as in nutrition.

For the AC-field activated swimmers, we use Janus particles of radius 1.00 ± 0.05 μm made of polystyrene (PS), coated with a thin layer of Cr and Au, 5 nm and 20 nm, respectively. They are activated *via* two different mechanisms based on the frequency of the applied field. In the low frequency regime (*f* < 100 KHz), the particles move towards the dielectric hemisphere due to induced charge electrophoresis (ICEP), resulting from the nonlinear electro-osmotic slip that occurs when an applied field acts on the ionic charge it induces around a polarizable surface. Whilst in the higher frequency regime, the dominating mechanism is self-dielectrophoresis (sDEP).^11^ The particles are suspended at sufficiently low concentration in deionized water with 1 mM MgSO_4_, and the resulting colloidal sample is placed between two glass slides coated with a 95 nm layer of conductive indium-tin-oxide (ITO) with a 10 nm layer of silica on top to prevent sticking.^19^ The glass slides are separated by a spacer of thickness 120 μm and connected to a function generator (GW Instek AFG-2005) as shown in Fig. 1(a), providing an AC electric field with constant amplitude of *V*_pp_ = 10 V and varying frequency in the range of 1 to 1000 kHz. Since the particles have a higher density than the solvent, they sediment to the bottom glass, and their motion is confined to the horizontal plane, except small thermally induced height fluctuations (see ESI†). The resulting active motion of the particles is followed with a bright-field microscope (ZEISS Axio Vert A1) equipped with a 63× objective with NA = 1.4, and recorded at a frame rate of 20 s^−1^. Particles are located with an accuracy of 25 nm in the image plane using image analysis,^20^ and their instantaneous velocities are determined from their displacement between frames by dividing by the fixed time interval Δ*t* = 0.05 s between images. A typical particle trajectory micrograph is shown in Fig. 1b, where sequential microscopy snapshots, 0.5 s apart, are overlaid over a ∼10 s time interval.

**Fig. 1:**
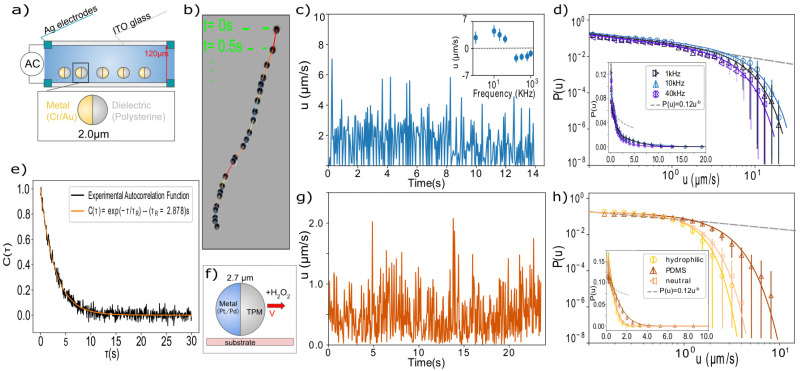
Intermittent motion of colloidal swimmers activated in an electric field by ICEP/sDEP and by catalysis of H_2_O_2_ (a) experimental setup for AC-field activation of Janus spheres. (b) Sequential microscope snapshots, 0.5 s apart, of a typical trajectory of an active Janus sphere. (c) Typical velocity trace of a single particle, showing rapid fluctuations in velocity magnitude over time. Inset: Average velocity as a function of electric field frequency, showing a switch of sign at 120 KHz. (d) Velocity distributions at three different frequencies in log–log representation (linear representation in inset). The velocities show a non-normal distribution, well fitted by a power-law with an exponential cut-off of the form *P*(*u*) ∝ *u*^−*b*^e^−*cu*^. (e) Autocorrelation function of the direction of motion. Experimental data averaged over the ensemble of particles (black) and exponential fit with time constant *τ*_R_ = 2.878 s (orange) (f) activation mechanism of the catalytic swimmers. (g) Velocity trace of a single catalytic particle, showing rapid fluctuations in velocity magnitude similar to panel (c). (h) Corresponding velocity distributions for measured for three different surfaces with disparate contact angles, fitted with truncated power-law distributions similar as in (d).

The advantage of the AC-field activation is that it allows tuning the magnitude of the active force *via* the frequency of the alternating field. Previous work has extensively explored the non-monotonic relation of the active force magnitude to frequency,^11^ passing through a cross-over frequency (COF) where the active force vanishes and switches sign. In agreement with these studies, we observe a similar average velocity-frequency relation, with velocity switching sign at ∼120 kHz, as shown in Fig. 1c (inset). Here, we have followed previous conventions to define the sign of the velocity to be positive when the particle is leading the motion with the dielectric hemisphere, and negative *vice versa*. Remarkably, when we resolve the motion with fine time resolution, we observe intermittency in the self propulsion of the particle even when it proceeds along straight paths, as clearly visible in the individual particle trajectory in Fig. 1b, and shown in the extracted velocity *versus* time in Fig. 1c. High velocities are followed by rapid slowing down.

To study this intermittency in more detail, we plot velocity distributions acquired for many particles in Fig. 1d. We do not consider orientation changes, since the characteristic time scale of orientation decorrelation, *τ*_R_ ∼ 2.9 s, which we measure through the orientational autocorrelation function of the ensemble (Fig. 1e and ESI†), is more than an order of magnitude larger than that of the velocity fluctuations. We observe that the distribution follows a power law, *P*(*u*) ∝ *u*^−*b*^, with exponent *b* = 0.45 ± 0.05 (dashed line), while the high velocity regime is dominated by an exponential cut-off, as confirmed by fitting over two orders of magnitude in velocity and more than 4 orders of magnitude in probability (solid lines). We also checked that the finite tracking accuracy did not affect the distribution, see ESI.† These truncated power-law velocity distributions are surprising and suggest some coupling between the ion-gradient mediated propulsion and the hydrodynamic drag with the wall.

To investigate the generality of this finding, we study a different active colloidal system of catalytic swimmers.^17,21^ The particles made from 3-(trimethoxysilyl) propyl methacrylate (TPM), with radius *R* = 1.35 μm (2.4% size polydispersity), were half-coated with 4.9 ± 0.2 nm Pt/Pd. The Janus spheres propel *via* preferential decomposition of H_2_O_2_ by the metallic hemisphere^22^ (Fig. 1d). A constant active force is achieved by control of the fuel concentration, while different propelling speeds are realized through different surface wetting properties. Similarly to the AC swimmers, particle sedimentation confines the motion to the horizontal plane. Besides the different swimming mechanism, the particles also differ in their cap-core mass ratios: while the Cr/Au coated polystyrene particles consisted of a light core and a heavy cap (see ESI†), these Pt/Pd particles consist of a heavy TPM core and a much lighter cap, giving rise to different torques exerted by gravity. Particle trajectories were followed with a 60× ELWD air objective (NA 0.7) on an inverted Nikon TI-E microscope at a frame rate of 19 s^−1^, typically within the hour after dispersing the colloids at dilute particle concentration ∼10^−7^ w/v) in deionized water containing 10% H_2_O_2_.

Despite the different driving mechanism and physical properties, we find very similar intermittency and power-law velocity distributions (Fig. 1g and h). We determine an exponent *b*′ = 0.46 ± 0.06 for all three surfaces, shown in Fig. 1h, indistinguishable within error bars from *b*. In the case here, the swimmers all experience the same propulsion force, set by the H_2_O_2_ concentration, but different hydrodynamic interaction with the substrate. The three different substrates have measured contact angles *θ* = 30° ± 3°, 51° ± 3° and 100° ± 3°, corresponding to a hydrophilic, a neutral, and a hydrophobic PDMS-coated glass surface. This leads to a shift of the exponential cutoff. Previous measurements showed that the average velocity scales as 〈*u*〉 ∝ (1 + cos(*θ*))^−3/2^.^17^ Here, we observe that the exponential cut-off shifts to the right with increasing contact angle, while the form of the distribution remains the same across the data.

The observation of power-law intermittency in two disparate systems suggests a general underlying mechanism based on the interplay of solute-driven propulsion^12–14^ and wall-induced hydrodynamic drag.^16^ To describe it, we construct a simple model for the dynamics of self-propelling particles, where the driving force generated by the particle-induced gradient is balanced by a drag force experienced by the moving particle, which scales linearly with velocity at low Reynolds numbers.^23^ The driving force is constant as long as the particle maintains the local gradient, which decays exponentially within an interfacial layer, *λ* ≪ *R*.^23^ For the AC system, the ionic gradient generated within the double layer decays within a characteristic length, the Debye length. Equivalently, for the catalytic swimmers, catalysis can occur when the H_2_O_2_ is in close proximity with the Pt cap, likewise defining a characteristic length at twice the size of the H_2_O_2_ molecule. At high velocities, however, the particle might run out of its gradient:^13,14^ due to solvent advection of the moving particle, the solute gradient building up by solute diffusion may not fully establish itself, thus reducing the propulsion force. This has been studied theoretically as a function of the Peclet number,^13,14^ relating the particle's swimming velocity, *u*, times radius, *R*, to the solute's diffusion coefficient, *D* (Pe = *uR*/*D*). At high Pe, when the advection effect due to the particle's motion becomes of the order of the solvent diffusion, the motion of the particle becomes wake-limited: the fast-moving particle runs ahead of its maximum gradient, thereby reducing its active force.

To account for this effect, we describe the velocity-dependent active force by considering a first-order negative loop relationship, *F*^eff^_a_ ∝ *F*_a_e^−(*u*/*u*_th_)^, where *F*_a_ is the maximum active force, which the particle experiences at rest, and *u* = d*x*/d*t* is the instantaneous velocity of the particle, and *u*_th_ ∝ *D*/*λ* is a characteristic velocity threshold proportional to the ratio of the relevant diffusion constant *D*, and length, *λ*. This ansatz reflects the fact that as the particle has moved a small distance d*x* out of the gradient, it will see an exponentially decreased solute concentration. The relation is of the form *F*^eff^_a_ ∝ e^−Pe^, with the aforementioned Pe number referring to the solute diffusion constant. The values of *u*_th_ for the AC-swimmers are calculated from the average diffusion coefficient (1.07 and 0.71 × 10^−3^ μm^2^ s^−1^ for sulfate and magnesium ions, respectively^24^), and the smallest Debye length of the 1 mM salt concentration (1.69 nm and 2.92 nm for the polysterene and Au hemispheres), giving *u*_th_ = 0.51 μm s^−1^. For the catalytic system, we used the diffusion coefficient 1.75 × 10^−4^ μm^2^ s^−1^ of H_2_O_2_ and the characteristic adsorption length *λ* = 5.95 × 10^−4^ μm, twice the size of an H_2_O_2_ molecule, yielding *u*_th_ = 0.34 μm s^−1^.

Concerning the drag force that the particles experience close to the wall, its magnitude depends on the slip length of the surface,^25,26^ which in turn depends on the contact angle *θ*.^27,28^ Previous theoretical work on the diffusio-osmotic motion of a fluid layer in the presence of a solute concentration gradient close to a wall,^16^ together with the slip length and its dependence on the contact angle^29^ suggests that the drag coefficient of a particle near the wall *c* ∝ (1 + cos(*θ*))^3/2^, as shown and experimentally confirmed in ref. 17. Due to inhomogeneities in the coating, and height fluctuations of the particle, the contact angle is expected to vary randomly within certain limits along the substrate. We model this effect by a spatially varying drag coefficient, *c*(*x*) ∝ (1 + cos(*θ*(*x*)))^3/2^, describing a random field of “obstacles” encountered by the particle, where hydrophilic patches will amplify the drag and hydrophobic patches will reduce it, creating an effective resistance to the motion.^25^ Together, the balance of active and drag forces leads to the equation of motion:1
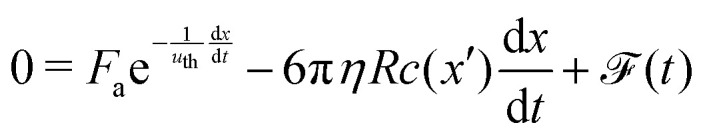
where *η* is the dynamic viscosity of the medium, *R* the particle radius, and ℱ(*t*) a random force term^30^ that accounts for the random fluctuations, and we have set the inertial component (left-hand side) to 0 since our system is overdamped. The velocity of fast moving particles will hence be limited by the diffusion-limited replenishment of the gradient (1st term), and the hydrodynamic drag (2nd). Eqn (1) is a non-linear ordinary differential equation, which can be solved numerically for *x* (see ESI†).

We employ eqn (1) to simulate velocity traces in one dimension using the calculated *u*_th_ value and the particle radii *R* of the two systems, and the viscosity of water *η* = 1 mPa s. To implement small fluctuations in the wetting angle, we sample a narrow Gaussian distribution, centered at the measured value of *θ*, after the particle has traversed a fixed length, resulting in a position-dependent *c*(*x*). The model uses a fixed temporal increment per step and a randomly generated 1-dimensional map of *c*(*x*) factors, where the time step is chosen small compared to the experimental resolution (0.5 μs). We can then differentiate |*x*| with respect to time to obtain velocity and construct a distribution.

Each of the two systems explores one of the two parameters, *F*_a_ and *c*(*x*). To find *F*_a_, we perform multiple runs, slowly increasing the active force, until good agreement with the experimental data is obtained. For the AC-experiments, this is done with a single *c*(*x*). For the catalytic swimmers, *vice versa F*_a_ was kept constant (due to the constant H_2_O_2_ concentration in the experiments) and *c*(*x*) was adapted to reflect the measured values of the wetting angle. The active force was selected such that the best fit was obtained for all three surfaces with respect to the experimental measurements.

Excellent fits are obtained with active forces *F*_a_ = 2.5, 3.5, and 1.2 pN at the frequencies of 1, 10, and 40 kHz, respectively, for the AC-field system, as well as with *F*_a_ = 0.5 pN and the experimentally measured contact angles *θ* for the catalytic swimmers, as shown in Fig. 2. Small deviations arise at the higher velocities, which could result from the small amount of experimental data at high velocities. Hence, in both cases, the dynamics described by eqn (1) are able to generate velocity distributions very similar to the experimental ones, capturing not only the power-law intermittency, but also the exponential cut-off, thus indicating the generality of the model.

**Fig. 2:**
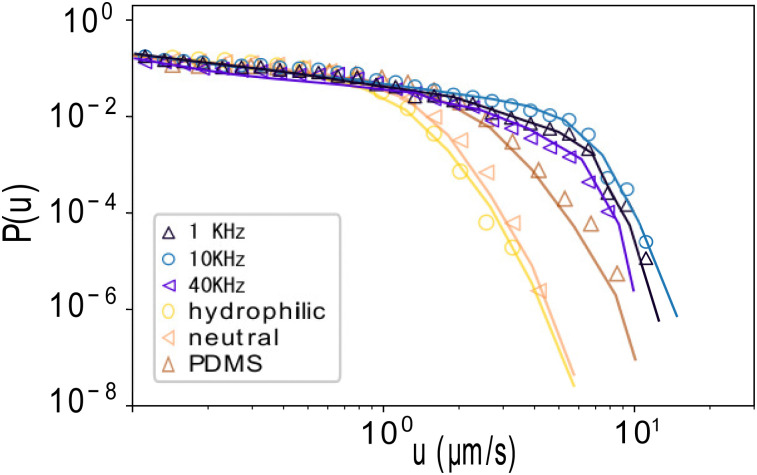
Model predictions. Velocity distributions of Ac-field and catalytic swimmers, for different frequencies and glass substrates respectively, in log–log representation for experimental (symbols) and model generated data (solid lines). The power law remains robust across both systems, different active forces, and different wetting angles.

To further highlight the generality of the underlying mechanism, we collapse the experimental data for all frequencies by re-scaling the velocity axis with *u*′. The collapsed data, together with the extracted scaling parameter *u*′ are shown in Fig. 3a. The extracted velocity scaling parameter *u*′ shows a frequency dependence very similar to that of the average velocity, see Fig. 1c inset, lending credence to our approach. We can similarly collapse the simulated data onto the same master curve and extract the active force *F*_a_ from the model. The latter shows a frequency dependence like the velocity, see inset.

**Fig. 3:**
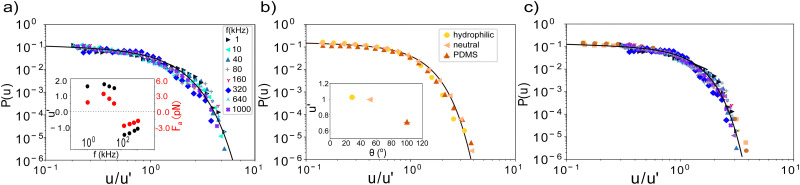
Generic mechanism. (a) Velocity distributions of the AC-field experiments, collapsed into a single master curve by scaling along the *x*-axis by *u*′. The scaling parameter *u*′ and corresponding *F*_a_ (inset) exhibit the same functional form as the average velocity in 1b (inset). (b) Velocity distributions of the catalytic swimmers, collapsed by scaling along the *x*-axis, by the ratios of the contact angles *θ* (inset). (c) Collapse of all data for both systems demonstrates general underlying mechanism.

A similar collapse is obtained for the three surfaces of the catalytic swimmers as shown in Fig. 3b. From the extracted velocity-scaling parameter, we can determine the contact angles of the substrates; using the neutral surface as reference, we then calculate the contact angles for the other two surfaces. We find values of 29° and 102° for the hydrophilic and PDMS surface respectively, in good agreement with the experimentally measured contact angles of 26° ± 3° and 100° ± 3°, supporting not only the relation proposed in ref. 17, but also highlighting the wetting-dependent drag force, as the effect is more pronounced at the high velocity regime.

We can also determine the three wetting angles directly from the model fits to the experimental velocity distributions. In this case, we allow the wetting angle *θ* to vary openly, whilst keeping *F*_a_ bound within a narrow limit defined by previous runs utilizing the knowledge that *F*_a_ has to be the same for all surfaces, as the fuel concentration is kept constant. Doing so, we find values of 29°, 49.5° and 102° for the hydrophilic, neutral, and PDMS surface from the best fit to the experimental data, which are in very good agreement with the experimentally measured values.

Finally, to show the robustness of our findings, we collapse all data of both systems in a single master curve as shown in Fig. 3c. The excellent collapse of all data in a single curve for both active swimmer systems, exploring the two different forces involved – active and friction force – highlights the general underlying mechanism. Specifically, the data suggest the same power-law exponent for both systems within error bars, and demonstrates the interplay between the hydrodynamic drag and velocity threshold as described by the model. The collapse demonstrates that changes in the magnitude of the active force as well as friction force *via* the contact angle affects the exponential cut-off, allowing the power law to extend to higher velocities for higher active forces or hydrophobic surfaces.

Our experiments and model provide a new perspective on the dynamics of active swimmers. Interesting intermittency arises due to (i) finite replenishment rate of the fuel gradient and (ii) varying surface drag fields due to the randomly fluctuating surface wetting properties. The resulting equation of motion balancing the active and hydrodynamic drag forces describes the experimentally observed intermittency and produces velocity distributions closely matching experimental measurements for two disparate systems.

The consistent power-law regime with robust exponent found for both systems suggests an underlying generic behavior. Interestingly, the two ingredients above are reminiscent of the basic local ingredients of depinning models,^31,32^ in which a local elastic driving force, set by the distance to a propagating front, together with a random field of obstacles leads to power-law velocity distributions with exponential cut-off. Yet, while the full description of depinning involves interaction of the different parts of the propagating front, this is not the case for colloidal swimmers at low density.

The observed large velocity spreads, as reported in ref. 9 and 11, may be directly related to the effects described in this Letter, namely the active force decay with velocity, and the hydrodynamic coefficient, which cannot be described by a Gaussian distribution. These results suggest further general implications for active swimmers. In fact, the observed intermittency is analogous to the intermittency expected for scallop-type swimmers in biological systems, as it contains all the necessary ingredients – non-reciprocal motion, broken symmetry, and a “relaxation” time before each propulsion action. While the scallop changes shape to achieve directional motion, active colloidal swimmers achieve the same result by changing the ionic or chemical concentration of their local surroundings.

## Conflicts of interest

There are no conflicts to declare.

## Supplementary Material

SM-020-D4SM00603H-s001
