# Supplementary material for: Power-law intermittency in the gradient-induced self-propulsion of colloidal swimmers
Source: Soft Matter. 2024 Jun 5;20(31):6103–8. doi: 10.1039/d4sm00603h (PMC11305149; doi:10.1039/d4sm00603h)
Supplement: SM-020-D4SM00603H-s001 [file SM-020-D4SM00603H-s001.pdf]

Electronic Supplementary Material (ESI) for Soft Matter.
This journal is © The Royal Society of Chemistry 2024

Power-law intermittency in the gradient-induced self-propulsion of colloidal swimmers - Supplementary Information

I. TRACKING AND LOCATING ERRORS

Tracking was done using the Python library Trackpy[1], which offers subpixel accuracy. In the AC field experiments, the pixel size is 136 nm, and the efficiency of the subpixel tracking is high due to the size of the particles and low signal to noise ratio of the images. We conservatively estimate a subpixel accuracy of 0.2 times the pixel size, corresponding to 25nm tracking error on each axis. We propagated the error for the x-y location using standard analysis $\Delta r = \sqrt{(\Delta x)^2 + (\Delta y)^2}$, and further by tacking the error for subsequent frames traking location $\Delta dr = \sqrt{(\Delta r_2)^2 + (\Delta r_2)^2}$, giving a total error in location of 70nm. For the temporal component we used the recorded frame error of 1ms provided by the microscope, to finally obtain a final error on the velocities, via standard error propagation, $\frac{\Delta u}{u} = \frac{\Delta dr}{dr} + \frac{\Delta dt}{dt}$. We note here that dt is aproximately constant at 50ms and results in a negligible error. The location error dominates, and remains small compared to the measured velocities. Furthermore, to check whether the aforementioned uncertainty affects our results, we artificially introduced Gaussian noise of width 25nm to our data.

As shown in Fig. S1, the addition of the noise affects only the low velocities, as is expected, and only minimally. The exponential cut-off is essentially identical in both cases for both examples. Therefore we conclude that the tracking error does not play any role in the observed intermittency.

To further ensure that the tracking error was not responsible for the observed behaviour, we also re-analyzed the data by only keeping every 2nd and every 4th frame from the analysis. This results in greater displacement, thus minimizing the effect of the tracking errors, shown Fig. S2. All distributions show the same power-law shape and exponential cut-off. confirming again that they are not significantly affected by noise. We re-scale the y-axis to highlight this behaviour, as removal of data points results in a different normalization constant.

Finally, we consider the effect of displacement due to diffusion. In order to ensure that it is not responsible for the observed behaviour, we analyze the velocities of significantly larger TPM particles of $r \sim 3\mu m$, which diffuse significantly slower. Due to their size, the activity is also reduced, however we still observe the same form of velocity distribution, shown in Fig. S3.

II. ORIENTATIONAL CHANGES

In the main manuscript we only make brief mention of the orientational changes, which do not affect the observed velocity fluctuations. This is because the time scale of orientational changes is much longer than that of the rapid velocity changes. Hence, the particles' intermittent motion is essentially observed along straight paths. To quantify the time scale of orientational decorrelation, we determine the angular autocorrelation function for an ensemble of particles using:

$$C(\tau) = \frac{< \theta(t)\theta(t+\tau) > - < \theta(t) >^2}{(< \theta(t)^2 > - < \theta(t) >^2)} \tag{1}$$

where θ is the orientation of the particle and τ is the lag time. The data is then fitted with an exponential of the form:

$$C(\tau) = exp(-\frac{\tau}{\tau_R}) \tag{2}$$

where τ_R is the decorrelation time. We obtain $\tau_R = 2.878$s from the best fit to the experimental data.

III. PARTICLE PHYSICAL PROPERTIES

In the main text, there are two separate set of experiments discussed. Each set was performed with different particles, which at first glance might appear similar, however actually present significantly different physical properties.

For the AC-field experiments, the particles consist of a polystyrene core, of density $\rho_{ps} = 1.05\ g/cm^3$, and a relatively thick cap of 25nm (5nm Cr, 20nm Au), of average density $\rho_{cap} = 17\ g/cm^3$ - taking into account the

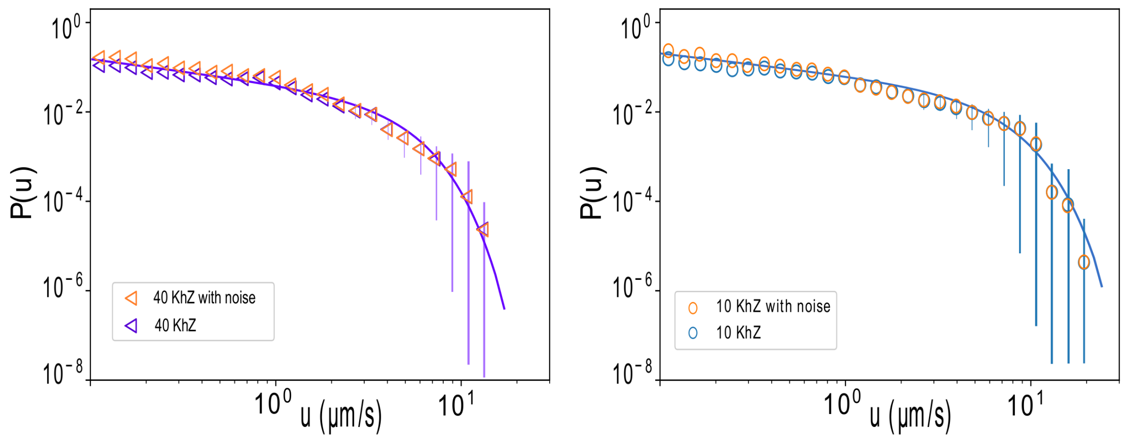

Figure S1: **Comparison of velocity distributions with added noise** Two examples of the velocity distrubutions for the 10 and 40 Khz, where a Gaussian noise of width 25nm was introduced in the tracking of each particle.

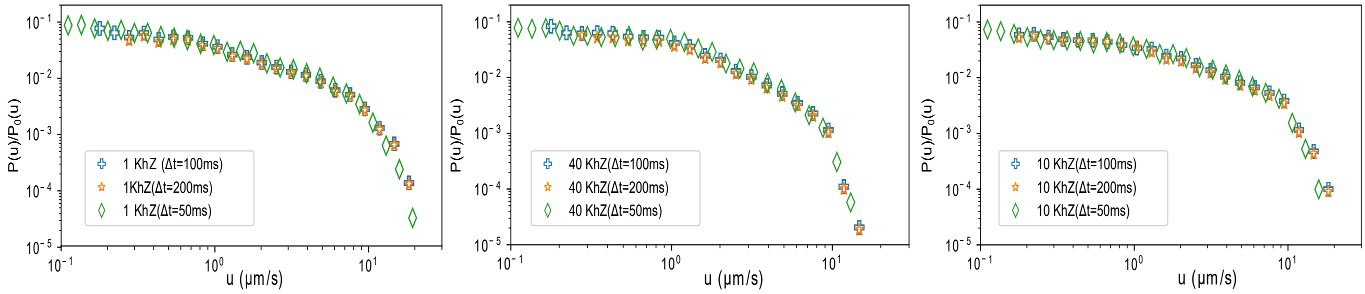

Figure S2: **Comparison of displacement distributions of full data vs reduced data for minimizing errors.** We reduce the data to minimize errors by removing every second (blue cross) and every fourth (orange star) frame from the data.We observe that the distributions maintain the same form, indicating that the observed behaviour is unaffected by the tracking error. For clarity we re-scale the y-axis for the reduced data by P_0, the original data probability.

lighter Cr of $\rho_{Cr} = 7.15\ g/cm^3$ and the heavier $\rho_{Au} = 19.3\ g/cm^3$. In water, this results in a core-to-coating mass ratio of:

$$\frac{m_{cap}}{m_{core}} = \frac{\frac{1}{2}\frac{4}{3}\pi(R^3_{capped} - R^3)(\rho_{cap} - \rho_{water})}{\frac{4}{3}\pi(R^3)(\rho_{ps} - \rho_{water})} = 12.32 \quad (3)$$

making the cap the main mass contributor. This has two main consequences: i) the particle is relatively heavy, experiencing a gravitational force of $F_g \sim 57.4fN$, confining the motion to be quasi-2D and ii) resulting in a high torque on the particle of $\tau \sim 3.2fN\mu m$, which we calculate by comparing the force exerted on each hemisphere. It is however well established, that the particle orientation is fixed with the broken symmetry aligned perpendicular to the surface, due to coupling of the surface and the electrical double layer of the particle [2].

In contrast, catalytic swimmers consist of a TPM core with density $\rho_{TPM} = 1.29\ g/cm^3$ and a much thiner cap of 5nm, made out of Pd/Pt with $\rho_{cap} = 21\ g/cm^3$. Using equation 1, the mass ratio is then:

$$\frac{m_{cap}}{m_{core}} = 0.38 \quad (4)$$

a difference of a factor of 50 compared to the PS particles. The particle remains heavy enough to be confined to its quasi-2D motion, with a gravitational force of $F_g \sim 39.3fN$, but experiences a significantly smaller torque of $\tau \sim 0.54fN\mu m$, almost an order of magnitude difference compared to the PS particles.

IV. EQUATION OF MOTION AND ITS SOLUTION

In the main text we introduced the force balance equation:

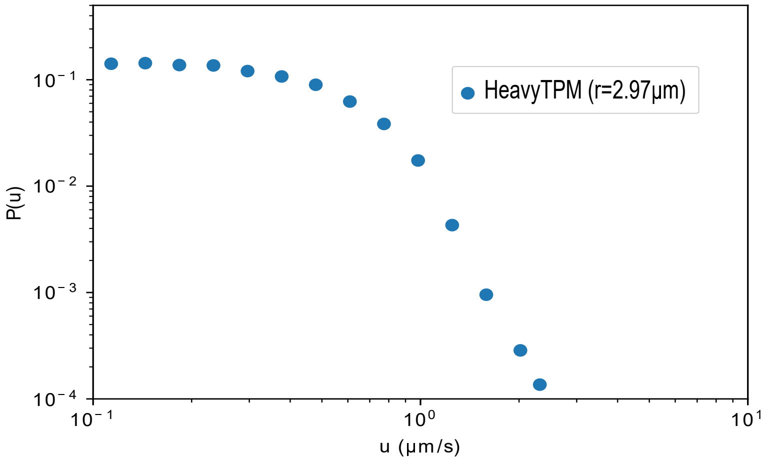

Figure S3: **Velocity distribution of heavier TPM particles, where motion due to diffusion is significantly reduced, indicating that activity gives rise to the observed behaviour.**

$$0 = F_a e^{-\frac{1}{u_{\mathrm{th}}}\frac{dx}{dt}} - 6\pi\eta R c(x')\frac{dx}{dt} + \mathcal{F}(t) \tag{5}$$

where we treat $\mathcal{F}(t)$ as an additive stochastic noise with 0 mean and delta-correlated autocorrelation. Equation (5) can be classified as a first-order nonlinear ordinary differential equation in $x(t)$, with standard solution:

$$x(t) = \int \left(u_{th} W \left(\frac{F_a exp(-\frac{\mathcal{F}(t)}{6\pi\eta Rc(x')u_{th}})}{6\pi\eta Rc(x')u_{th}} \right) + \frac{\mathcal{F}(t)}{6\pi\eta Rc(x')} \right) dt \tag{6}$$

which contains the log-product function W. The log-product function has multiple branches and can take any complex argument, however for each argument there is only one real branch[3]. Eq. 4 is in the most convenient form for numerical integration, however it remains rather obscure in terms of the physics. To illustrate better the physics contained in this equation we can introduce the instantaneous velocity u_i into the equation and re-write as:

$$x(t) = \int_0^{t'} \left(u_{th} W \left(\frac{F_a}{F_{drag}} \frac{u_i}{u_{th}} exp(-\frac{u_i}{u_{th}} \frac{\mathcal{F}(t)}{F_{drag}}) \right) + \frac{\mathcal{F}(t)}{6\pi\eta Rc(x')} \right) dt \tag{7}$$

where we have chosen a t' small enough such that the velocity remains constant in the interval. Here we have introduced u_i and $F_{drag} = 6\pi\eta Rc(x')u_i$.

Using Eq. 6, we can execute a numerical integration for our model by choosing a fixed time step, which allows us to re-write the diffusion contribution to displacement as $(2D\Delta t)^{1/2}\mathcal{N}(0,1)$, where $\mathcal{N}(0,1)$ is a Gaussian distribution with mean 0 and standard deviation 1. Hence we can use the time series:

$$X_{t+\Delta t} = X_t + u_{th} W \left(\frac{F_a exp(-\frac{1}{u_{th}}\sqrt{\frac{2k_BT}{6\pi\eta Rc(x')}}\mathcal{N}(0,1)}{6\pi\eta Rc(x')u_{th}} \right) \Delta t + \sqrt{2D\Delta t}\mathcal{N}(0,1) \tag{8}$$

where we have used a small $\Delta t = 0.5\mu s$, such that $\sqrt{2D\Delta t} << \lambda$, the interfacial layer. This is done to ensure that within each time step, $c(x')$ can be considered constant.

We note here that the log-product function contains the competition between the active force and the drag force, as well as the scaling defined by the particle-solute interaction u_{th}. We can explore the limits of the log product function to gain further insight. When $F_a = 0$ and $W(0) = 0$, we retrieve the pure diffusion case, as expected. When increasing F_a, initially W increases relatively rapidly, but logarithmically slows down, as shown in Fig. S4. This can be understood as a saturation of the activation mechanism. It is important to note here that beyond a certain F_a, we expect the assumption for low Re numbers to collapse and the analysis to become invalid. We further note that the

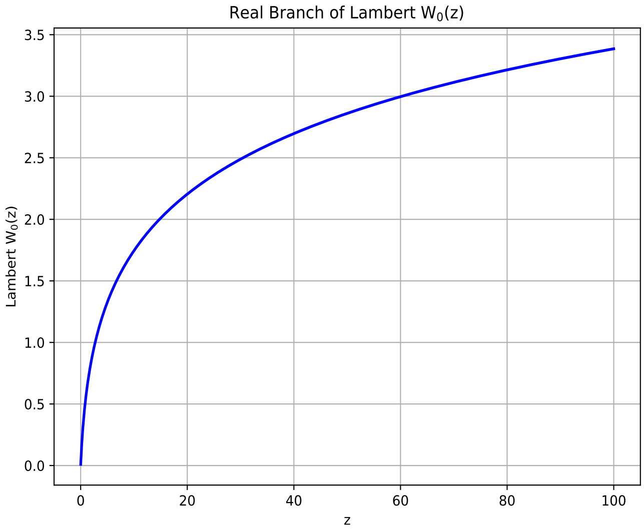

Figure S4: **Real branch of the log-product function for values ranging from 0 to 100.** We initially see a rapid increase, followed by logarithmic slow down. The x-axis corresponds to the argument of the log-product function as defined in Eq.(8)

argument of the W function scales as $\sim e^{-\frac{1}{u_{th}}}$, indicating that a larger interfacial layer, or a faster diffusing solute, can enhance activity, consistent with predictions [4, 5].

[1] D. Allan and T. Caswell, *Trackpy* (2017).
[2] A. Boymelgreen, G. Yossifon, and T. Miloh, Langmuir **32**, 9540 (2016), ISSN 0743-7463, URL `https://doi.org/10.1021/acs.langmuir.6b01758`.
[3] D. Veberič, Computer Physics Communications **183**, 2622 (2012), ISSN 0010-4655, URL `https://www.sciencedirect.com/science/article/pii/S0010465512002366`.
[4] J. F. BRADY, Journal of Fluid Mechanics **667**, 216–259 (2011).
[5] E. Yariv and S. Michelin, Journal of Fluid Mechanics **768**, R1 (2015).
